# Personalized practice dosages may improve motor learning in older adults compared to “standard of care” practice dosages: A randomized controlled trial

**DOI:** 10.3389/fresc.2022.897997

**Published:** 2022-08-03

**Authors:** Geneviève N. Olivier, Leland E. Dibble, Serene S. Paul, Keith R. Lohse, Christopher S. Walter, Ryan J. Marker, Heather A. Hayes, K. Bo Foreman, Kevin Duff, Sydney Y. Schaefer

**Affiliations:** ^1^Department of Physical Therapy and Athletic Training, University of Utah, Salt Lake City, UT, United States; ^2^Center on Aging, University of Utah, Salt Lake City, UT, United States; ^3^Discipline of Physiotherapy, Faculty of Medicine and Health, Sydney School of Health Sciences, University of Sydney, Sydney, NSW, Australia; ^4^Department of Health-Kinesiology-Recreation, University of Utah, Salt Lake City, UT, United States; ^5^Program in Physical Therapy and Department of Neurology, Washington University School of Medicine in St. Louis, St. Louis, MO, United States; ^6^Department of Physical Therapy, University of Arkansas for Medical Sciences, Fayetteville, AR, United States; ^7^Department of Physical Medicine and Rehabilitation, University of Colorado Anschutz Medical Campus, Aurora, CO, United States; ^8^Department of Neurology, University of Utah, Salt Lake City, UT, United States; ^9^School of Biological and Health Systems Engineering, Arizona State University, Tempe, AZ, United States

**Keywords:** overpractice, practice dosage (repetition), motor learning, personalized medicine, postural control, aging, older adults, motor rehabilitation

## Abstract

Standard dosages of motor practice in clinical physical rehabilitation are insufficient to optimize motor learning, particularly for older patients who often learn at a slower rate than younger patients. Personalized practice dosing (i.e., practicing a task to or beyond one's plateau in performance) may provide a clinically feasible method for determining a dose of practice that is both standardized and individualized, and may improve motor learning. The purpose of this study was to investigate whether personalized practice dosages [*practice to plateau* (PtP) and *overpractice* (OVP)] improve retention and transfer of a motor task, compared to *low dose* [LD] practice that mimics standard clinical dosages. In this pilot randomized controlled trial (NCT02898701, ClinicalTrials.gov), community-dwelling older adults (*n* = 41, 25 female, mean age 68.9 years) with a range of balance ability performed a standing serial reaction time task in which they stepped to specific targets. Presented stimuli included random sequences and a blinded repeating sequence. Participants were randomly assigned to one of three groups: LD (*n* = 15, 6 practice trials equaling 144 steps), PtP (*n* = 14, practice until reaching an estimated personal plateau in performance), or OVP (*n* = 12, practice 100% more trials after reaching an estimated plateau in performance). Measures of task-specific learning (i.e., faster speed on retention tests) and transfer of learning were performed after 2–4 days of no practice. Learning of the random sequence was greater for the OVP group compared to the LD group (*p* = 0.020). The OVP (*p* = 0.004) and PtP (*p* = 0.010) groups learned the repeated sequence more than the LD group, although the number of practice trials across groups more strongly predicted learning (*p* = 0.020) than did group assignment (OVP vs. PtP, *p* = 0.270). No group effect was observed for transfer, although significant transfer was observed in this study as a whole (*p* < 0.001). Overall, high and personalized dosages of postural training were well-tolerated by older adults, suggesting that this approach is clinically feasible. Practicing well-beyond standard dosages also improved motor learning. Further research should determine the clinical benefit of this personalized approach, and if one of the personalized approaches (PtP vs. OVP) is more beneficial than the other for older patients.

## Introduction

*Motor learning* is a set of practice-related internal processes leading to a relatively permanent change in the ability to perform a motor skill (1). But the optimal amount, or dose, of task practice that is needed for a person to learn a motor skill remains unclear. Studies suggest that “more is better” in general (2–4), but how much more? Dosages of motor practice in clinical rehabilitation are commonly quantified in terms of time (e.g., a 45-min therapy session). However, observational studies demonstrating that the amount of actual task practice is very low in standard clinical care (5) have resulted in a shift, such that practice dosage has more recently been quantified as the number of repetitions performed (4–6).

Animal studies of stroke rehabilitation have demonstrated motor skill recovery that is substantially better than what is typically seen in humans (3, 7–9). Although many factors contribute to this phenomenon, the dose of practice implemented is a critical difference between neurologic rehabilitation in animal models vs. that in humans (3–5). Animal studies incorporate very high repetitions of daily practice (hundreds to thousands) (4, 5, 10, 11), whereas observational studies of human neurologic rehabilitation provided in clinics show practice repetitions primarily below 100 (and sometimes below 10) (4–6, 10, 11), resulting in frequent under-dosing. Under-dosing of practice is also present in human motor learning research [e.g., (12)], and may in part explain their null findings.

While data suggest that standard clinical dosages typically yield inadequate practice amounts to result in motor learning, the optimal number of repetitions needed to obtain a clinical benefit is not clear. What is clear, however, is that motor skill acquisition is quite variable between individuals (13, 14), as are time scales of learning (15, 16). Because of this inter-individual variability in motor skill acquisition and learning, it is unlikely that there is one optimal practice dose, but rather, motor practice dosages should be more personalized. One method for doing so could be to use a personal plateau in performance during practice (henceforth termed *practice plateau*) as the “threshold” for dosing motor task practice. Typically, performance on a motor task improves with practice (e.g., less error or faster speed) until it begins to stabilize (17–19) (i.e., *plateau*). Additional practice provided beyond when an individual reaches a practice plateau is termed *overpractice*. Practicing to or beyond the point where task performance has plateaued may improve learning (15, 20, 21), and would provide a method for dosing practice that is both standardized and individualized.

Motor learning research has primarily been performed in healthy young people, leaving large gaps when it comes to older adult and clinical populations (22). However, motor learning principles are especially relevant to older adults and people with neurological disorders, because both groups have a critical need to learn or relearn motor skills to maintain independent function. Because older adults use physical rehabilitation services at higher rates than younger adults, rehabilitation must be optimized for older adults (23) rather than using a “one-size-fits-all” approach. Shedding light on the optimal amount of practice needed to obtain retention and transfer will improve training prescription in physical rehabilitation settings.

Thus, the purpose of this study was to examine the premise that personalized practice dosages (i.e., practicing to and beyond one's personal practice plateau) improve motor learning of a standing postural task in community-dwelling older adults with a range of balance abilities. The primary hypothesis was that practice dosages based on individual skill acquisition patterns would improve learning compared to lower practice dosages that mimic the clinical standard of care. Learning was measured as: (1) *retention* [i.e., performance on the practiced task after a period of no practice (1)], and (2) *transfer* [i.e., change in proficiency on an untrained task as a result of experiencing the practiced task (24)].

## Materials and methods

### Design

This pilot randomized controlled trial (NCT02898701, clinicaltrials.gov) was approved by the University of Utah (Salt Lake City, UT, USA) Institutional Review Board. Before enrolling, participants provided written informed consent. Study appointments were performed in research labs at the University of Utah. A trained assessor performed baseline assessments, after which a sealed envelope was opened to determine group assignment to one of three groups. Blocked randomization was used to ensure equal group size (15 per group).

### Participants

Between August 2018 and March 2020, community-dwelling older adults were recruited from the Salt Lake City metropolitan area. Eligible participants were aged 60–90 years. Exclusion criteria included: cognitive impairment (Montreal Cognitive Assessment score <26) (25), uncorrected visual impairment, non-English speaking, acute medical conditions, or any other conditions affecting mobility or balance to the extent that it impacted the ability to perform the motor task (e.g., arthritic, orthopedic, metabolic, vestibular). Baseline demographics (Table 1), cognitive assessment (Montreal Cognitive Assessment), and gross motor function including self-selected and fast gait speeds *via* the 10 Meter Walk Test (26, 27) and the mini-Balance Evaluation Systems Test (28), were collected to characterize the sample's cognitive and motor function. Trained study staff supervised all practice sessions and performed retention and transfer tests. Because sleep can impact motor consolidation and learning (29), immediately following randomization, participants were instructed to keep a sleep diary in which they logged their daily minutes of sleep, starting the morning of their first day of motor task practice and ending on the final test day.

**Table 1:** Participant characteristics by group.

**Outcome**	**LD (*n* = 15)**	**PtP (*n* = 14)**	**OVP (*n* = 12)**	**All (*n* = 41)**
Age (years)	69.1 (5.8)	68.3 (6.2)	69.3 (5.5)	68.9 (5.7)
	[65.9, 72.3]	[64.7, 71.9]	[65.8, 72.8]	[67.1, 70.7]
Sex (F)	10 (66.7%)	8 (57.1%)	7 (58.3%)	25 (61.0%)
Race/Ethnicity: Asian	1 (6.7%)	0 (0%)	0 (0%)	1 (2.4%)
Race/Ethnicity: Native Hawaiian or other Pacific Islander	0 (0%)	0 (0%)	1 (8.3%)	1 (2.4%)
Race/Ethnicity: White	14 (93.3%)	14 (100%)	11 (91.7%)	39 (95.1%)
Height (in)	64.7 (4.9)	66.7 (3.7)	66.7 (3.4)	65.9 (4.1)
	[62.0, 67.4]	[64.6, 68.8]	[64.6, 68.8]	[64.6, 67.2]
MoCA (0-30)^*^^†^	28.3 (1.2)	27.9 (1.3)	28.1 (1.2)	28.1 (1.2)
	[27.6, 29.0]	[27.2, 28.6]	[27.4, 28.8]	[27.7, 28.5]
Self-selected gait speed (m/s)^*^	1.33 (0.18)	1.36 (0.21)	1.32 (0.16)	1.34 (0.18)
	[1.23, 1.43]	[1.24, 1.48]	[1.22, 1.42]	[1.28, 1.40]
Fast gait speed (m/s)^*^	1.81 (0.28)	1.96 (0.24)	1.82 (0.24)	1.86 (0.26)
	[1.65, 1.97]	[1.82, 2.10]	[1.67, 1.97]	[1.78, 1.94]
Mini-BEST^*^	24.1 (2.7)	23.7 (2.0)	23.1 (2.5)	23.7 (2.4)
	[22.6, 25.6]	[22.6, 24.8]	[21.5, 24.7]	[22.9, 24.5]
Average sleep between sessions	399.3 (51.9)	420.5 (47.6)	414.5 (49.0)	411.0 (49.27)
(minutes)	[370.5, 428.1]	[393.0, 448.0]	[383.3, 445.7]	[395.4, 426.6]
Number of practice trials performed	6 (0)	37.6 (17.2)	61.2 (18.4)	32.9 (26.6)
	[6.0, 6.0]	[27.7, 47.5]	[49.5, 72.9]	[24.5, 41.3]
Remained blinded to repeating sequence	9 (60%)	9 (64%)	9 (75%)	27 (65.9%)
Retention interval^‡^: 2 days	10 (66.7%)	6 (42.9%)	5 (41.7%)	21 (52.5%)
Retention interval^‡^: 3 days	5 (33.3%)	5 (35.7%)	5 (41.7%)	15 (36.6%)
Retention interval^‡^: 4 days	0 (0%)	2 (14.3%)	2 (16.7%)	4 (9.8%)

Data presented as mean (SD) [95% confidence interval] or *n* (%).

* Higher score indicates better performance.

† <26 typically indicates cognitive impairment.

‡ Retention Interval: number of days of no practice prior to retention test day. There was also a protocol deviation for 1 of the PtP participants, who became ill and had a 13-day retention interval (not shown above).

LD, Low Dose of practice group; PtP, Practice to Plateau group; OVP, Overpractice group; MoCA, Montreal Cognitive Assessment; mini-BEST, mini-Balance Evaluation Systems Test.

### Motor task

Using a serial reaction time task (SRTT) (30–32), participants mapped a visuospatial stimulus to a corresponding response location. The stimulus was presented in a string of either random or repeating sequences. Learning of the random and repeating sequences is thought to reflect *general learning* and *repeated-sequence learning*, respectively (20). The most common SRTTs used consist of seated upper extremity reaching and pointing, although recent studies have used a standing SRTT, in which participants step to the target instead of reaching to it (20, 33, 34). We selected this standing SRTT task because it has been shown to be a feasible and efficacious paradigm for measuring motor learning (33), specifically examining learning of stepping, which is a salient aspect (35) of the anticipatory postural adjustments necessary for functional standing and stepping.

**Figure 1:**
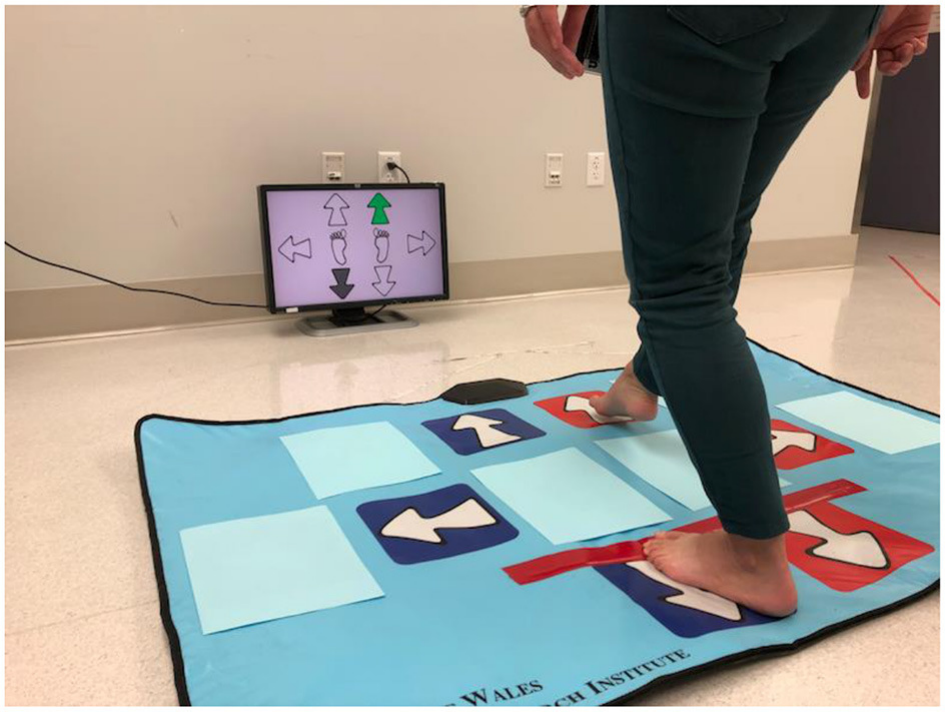
An image of the instrumented step mat, with a person mid-step. Instructions provided to participants throughout the study included: **At the start of Day 1**: *Start with one foot on each of the arrows at the back of the mat, behind the red tape. That's your START POSITION. When one of these arrows* (POINT) *turns green, step to the corresponding target on the mat as fast as you safely can, then return that foot to your start position. Next, one of the other arrows will turn green (POINT), and you will step to that arrow's corresponding target as fast as you safely can. Each time you make contact with a target, the arrow on the screen should change colors. If it does not change colors, that means you didn't make sufficient contact with the target, so you must try again. Whenever you step, always use the foot that's closest to the target, instead of crossing your feet. Also, please keep your hands by your sides, or on your hips (not crossed, or in your pockets, etc); and please don't talk while you're stepping. Lastly, remember to keep your feet behind the red tape when you're in the start position. You will continue stepping until the screen says that the trial is over. You will get a short standing rest break after every trial, and a 4-minute seated rest break after every 6 trials. Remember, your goal is to step to the target as fast as you safely can. Do you have any questions? Ready?* **At the end of each Block**: *You have completed _____* (1, 2, etc) *blocks of training. You now have a 4-minute rest break. Please take a seat. For that block, on average it took you ____ seconds to complete each step. Your goal is to take even less time on this next block, if you can safely do so*. **At the end of each Seated Rest Break**: *Your break will end in 30 seconds. Please stand up and return to your start position. Remember, your goal is to step to the target as fast as you safely can, attempting to beat your previous score if possible*. **At the start of each Day following Day 1**: *Just like yesterday, your goal is to step to the target as fast as you safely can*. *Your best score yesterday was ____ seconds. The best score you've ever gotten was _____ seconds. Your goal is to go even faster during today's training, if you can safely do so. Lastly, remember to keep your feet behind the red tape when you're in the start position. Do you have any questions? Ready?* **At the start of the Test for Explicit Awareness**: *You are done with that task. Now, you will stand on the mat and go through 10 shorter trials at your comfortable pace. Your stepping speed is not important for this part. At the end of each trial, you will say “Yes, I recognize that sequence from my training sessions” or “No, I don't recognize that sequence from my training sessions.” There may be times when you feel unsure, but you must commit to an answer of YES or NO… just make your best guess*.

A description of this standing SRTT has been published in detail previously (33). To summarize, participants stood on an instrumented step mat (33, 36) and were instructed to step as fast as they safely could to the corresponding position on the mat in response to a visual stimulus presented on a computer screen (Figure 1). The mat's size, design, and orientation were identical for all participants. Participants stepped from one of the two “home” pressure pads (side-by-side at the back of the step mat) to one of the four targets (one each to the right and left of “home,” and two side-by-side directly in front of “home”) using the ipsilateral foot relative to the target location (e.g., the left foot stepped to targets to the left or left front of “home”). When the appropriate amount of force was applied to the correct target (minimum 66.4 Newtons, or 6.8 kg), the stimulus dimmed on the computer screen and participants returned their stepping foot to “home.” Participants had to apply enough force to the correct target with one foot, and then apply enough force to both home pressure pads (with one foot on each home pressure pad), before the subsequent stimulus would appear. *Response time* was collected and defined as the time in seconds from stimulus presentation to foot pressing on target (33). If a participant stepped to the wrong target, the visual stimulus would remain on the computer screen until the participant completed the step correctly (i.e., to the correct target, and with the requisite amount of force). The participant's response time for that step would be prolonged with this type of error; thus, improved response times reflect improvement in both speed and accuracy.

Each trial consisted of two 12-step sequences: one random, one repeating, although participants were blind to the presence of the repeating sequence. Within each trial, these sequences were presented in a random order. To ensure equal representation of the four targets, each target was presented three times during every 12-step sequence. One practice trial contained two consecutive 12-step sequences (one random, one repeating) presented in random order. One block of practice contained six trials (i.e., 144 total steps, half of which were made up of the random sequences while the other half were made up of the repeating sequence). A 25-s standing rest break was provided between trials, and a 4-min seated rest break between blocks. In order to optimize participants' learning of the motor task, intermittent written and verbal feedback was provided during seated rest breaks, in the form of median response time^1^ for the preceding block (37–39).

**Figure 2:**
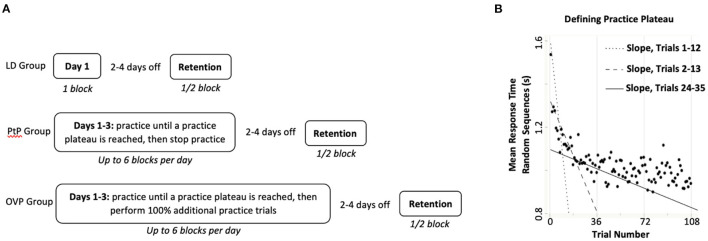
**(A)** Practice and retention testing timeline for each group. LD, Low Dose group; PtP, Practice to Plateau group; OVP, Overpractice group. Participants randomly assigned to the LD group completed 1 block of 6 trials of task practice over one training day, while those in the other two groups performed up to 6 blocks of practice per day for several days. Specifically, people in the PtP group practiced the task until they achieved a practice plateau (between 1 and 3 training days), at which point they stopped practicing; participants in the OVP group performed 100% additional practice trials after reaching a practice plateau (between 1 and 3 total training days). The retention test occurred following 2–4 days of no practice. **(B)** A hypothetical illustration of how iterative slopes are calculated for a participant, in order to determine their practice plateau. A scatterplot (solid circles) of mean response time on the random sequences of the SRTT (*y*-axis) is shown as a function of practice trial number (*x*-axis). Using Ordinary Least-Squares Regression, a slope is calculated for Trials 1–12. Iterative slopes are then calculated immediately after each additional trial is completed, for Trials 2–13, Trials 3–14, Trials 4–15, Trials 5–16, etc. Generally, successive slopes become less steeply negative (i.e., flatter) as the participant practices, as illustrated by the three exemplar slope lines shown. Iterative slopes continue to be calculated until the participant is found to have reached a practice plateau. A slope of >-0.001 was considered to be little-to-no improvement on the task and each participant was considered to have reached a practice plateau after custom-coded software calculated seven consecutive slopes greater than threshold.

One complete day of practice included six blocks of practice, lasting ~90 min. After completing their assigned practice dose, participants had 2–4 days of no practice before returning on post-practice days 3–5 for a retention test consisting of three trials of the SRTT (Figure 2A) and a post-test of the transfer task. Study staff was not blinded to group assignment during retention test and post-test. A retention interval of 2–4 days was allowed due to participant scheduling challenges, which sometimes prohibited them from returning after exactly 2 days of no practice.

### Group assignment

After collecting demographic information as well as cognitive and gross motor function, we randomly assigned participants to one of three groups: *low dose* (LD) practice (one block of six trials of task practice over one training day), *practice to plateau* (PtP; practice until reaching one's own individual practice plateau, then stop practicing), or *overpractice* (OVP; practice until reaching one's own individual practice plateau, then perform 100% more practice trials) over three training days (Figure 2A).

### Defining practice plateau

For this study, faster response times during practice demonstrated improvement in skilled performance of the SRTT. Differentiating between the two personalized practice groups (PtP, OVP) required identifying the point at which each participant's personal practice plateau was reached.

Briefly, plateau was identified by iteratively calculating response time performance slopes in real time. A custom-written algorithm (custom LabVIEW software, National Instruments, Austin, TX) first calculated a mean response time on the random sequence steps for each trial [automatically removing gross outliers (>5 s) from the raw data, as large outliers caused instability in the algorithm]. Immediately upon completion of Trial 12, the algorithm used Ordinary Least Squares regression to calculate a performance slope that included the current and preceding 11 trials (12 total). This was repeated for each subsequent trial using a sliding window of the last 12 consecutive trials (e.g., trials 2 through 13, trials 3 through 14, etc.). Because response time tends to improve rapidly early on, these slopes tended to be very negative (representing significant improvement) during early practice, and level-off closer to zero (i.e., less negative, representing less improvement) later in practice. A slope of >-0.001 was considered to represent little-to-no improvement. A *practice plateau* was identified after seven consecutive slopes of >-0.001 (Figure 2B).

Practice for the PtP group was discontinued immediately upon reaching this calculated practice plateau. Practice for the OVP group continued until 100% more practice trials were performed after reaching the calculated practice plateau. For example, if plateau was reached by an OVP participant at Trial 25, they would then perform 25 additional practice trials, for a total of 50 trials.

### Testing for explicit awareness

Although participants were blinded to the presence of the imbedded repeating sequence, it was possible to become explicitly aware of it due to repetitive exposure to the task. To control for this potential threat to internal validity, explicit awareness of the repeating sequence was tested. Following the retention test each participant performed ten 12-step sequences presented in random order, half of which consisted of the repeating sequence while the other half consisted of novel random sequences. After experiencing each sequence, participants were asked whether they recognized it from the practice sessions. Participants were considered to have gained explicit awareness of the repeating sequence if they were able to respond correctly to each type of sequence at better than a chance rate (i.e., correctly identified the random sequences >50% and the repeating sequence >50%) (40).

### Data reduction and statistical analyses

#### Power analysis

The SRTT selected for this study has demonstrated a between-group effect size of 0.27 (Cohen's *f*^2^) for general learning in pilot data. *A priori*, we powered the study to detect the omnibus effect of group based on this estimate. Assuming alpha = 5% and three total predictors [one controlling for pretest and two additional predictors to test the omnibus effect of group (i.e., PtP and OVP groups compared to LD)], we estimated that a total sample of 39 participants would be necessary to detect an omnibus effect of group with 80% power (41). Allowing for attrition, we sought to recruit 45 total participants.

#### Data reduction

Visual inspection and cross-referencing with staff notes taken during practice sessions revealed some outlying response time data points that were related to technical difficulties with administering the SRTT (e.g., step mat time-outs and computer crashing). In the absence of technical errors, response times were consistently <2.5 s. Thus, to eliminate equipment and computer errors while preserving steps that included true participant errors, extreme outliers (response time >2.5 s^2^) were excluded from analysis, resulting in 1% of SRTT data being removed and treated as missing. Descriptive statistics of participants' baseline characteristics were obtained using JMP Pro 14 (SAS Institute, Cary, NC). All analyses of outcomes were conducted in R (v3.4.1; R Core Team, (42)). Alpha levels were set at 0.05.

#### Dependent variables

*Pretest performance* was defined as mean response time on the first three trials of the first day of practice. *Retention performance* served as the primary outcome for this study and was defined as mean response time during retention testing. For both timepoints, calculations of mean response times were performed separately for the random and repeating sequences. The primary dependent variables were *general learning* (retention performance^3^ on the random sequences) and *repeated-sequence learning* (retention performance on the repeating sequence) (20, 33). The secondary dependent variable was performance time on a transfer task, used to test the extent to which proficiency on a task changes as a result of practicing another task (24). The Four Square Step Test (44) served as the transfer task; it was administered prior to starting SRTT practice (pretest) and again immediately following the SRTT retention test (posttest) according to published instructions (44), such that faster times indicate better performance.

#### Retention and transfer analysis

We performed two multiple regression models for retention of each type of sequence: random and repeating. The first model tested the omnibus effect of group and included pretest performance on the SRTT and group assignment (with LD serving as the reference). The second model added explicit awareness, and the group-by-explicit awareness interaction, to determine whether becoming explicitly aware of the repeating sequence impacted learning. *Post-hoc t*-tests were performed if the omnibus effect of group was statistically significant. To test *transfer of learning* (posttest performance on the Four Square Step Test), the regression model tested the omnibus effect of group, and included pretest performance on the Four Square Step Test as well as group assignment (with LD serving as the reference). If the omnibus effect of group was statistically significant, then *post-hoc t*-tests were performed.

*Post-hoc* testing was performed to determine if the results were more strongly related to the experimental condition (i.e., group assignment) or to the absolute number of trials performed by each participant. The LD group was excluded from these *post-hoc* tests, as these participants performed exactly six trials of the SRTT. Separate regression models were used for each dependent variable (*general learning, repeated-sequence learning*, and *transfer of learning*). The model included pretest performance on that variable, total number of trials performed by each participant, and group assignment (PtP or OVP). Because number of trials and group assignment are highly correlated, the effect of number of trials was examined first and then the factor of group was added to determine if the effect of number of trials was attenuated.

## Results

### Participant flow and characteristics

**Figure 3:**
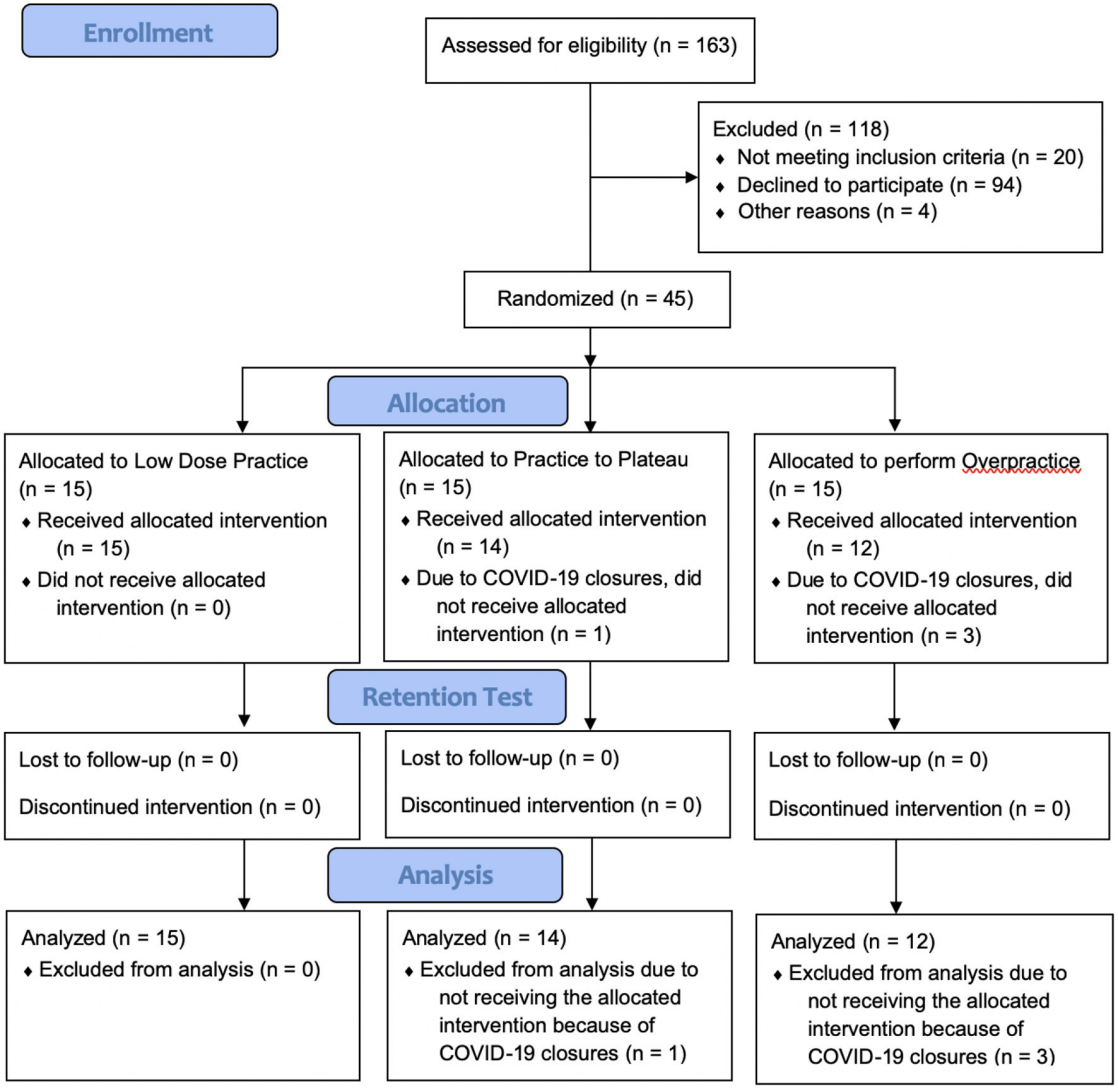
Participant flow through the pilot randomized controlled trial.

Forty-five adults were enrolled; however, four (8.8%) were unable to participate in their allocated intervention due to nationwide closures related to the COVID-19 pandemic (Figure 3). Participant characteristics and results were calculated for the remaining 41 participants (25 female; mean age 68.9 years). Age, height, Montreal Cognitive Assessment scores, self-selected and fast gait speeds, and mini-Balance Evaluation Systems Test scores do not appear noticeably different at baseline (Table 1). Reported amounts of sleep between practice sessions also do not appear different between groups (Table 1). Motor and cognitive function were generally within normal limits. However, mini-Balance Evaluation Systems Test scores indicated balance impairment within the cohort, as evident by an overall mean score of 23.7/28 [just below the minimal clinically important difference (i.e., 4 points) compared to a perfect score (45)], with nine participants (22%) scoring ≤ 21/28 [cutoff score for postural response deficits (46)]. Frequency counts for the number of participants who had each retention interval length (i.e., 2, 3, or 4 days of no practice before returning for retention test day) are also shown in Table 1. There was one protocol deviation, in which 1 participant in the PtP group became ill after their final training day, resulting in a 13-day retention interval (not included in Table 1). Overall, median retention interval length was as follows: all participants 2 days, LD Group 2 days, PtP Group 3 days, OVP group 3 days.

### Number of practice trials performed and explicit awareness

All participants (*n* = 41) completed their entire assigned dose of practice (Table 1) without any adverse events. All in the LD group (*n* = 15) completed exactly six practice trials. Participants in the PtP group (*n* = 14) stopped practicing immediately after reaching their personal practice plateau, which took an average of 37.6 (range 18–74) trials. Those in the OVP group (*n* = 12) reached plateau after an average of 30.6 (range 18–43) trials, and then performed 100% more practice trials, eventually stopping practice after an average of 61.2 trials (range 36–86). Over the course of practice, 60, 64, and 75% of participants in the LD, PtP, and OVP groups, respectively, remained blinded to the repeating sequence throughout the entire study, despite repetitive exposure to it.

### Learning outcomes

#### General learning

**Figure 4:**
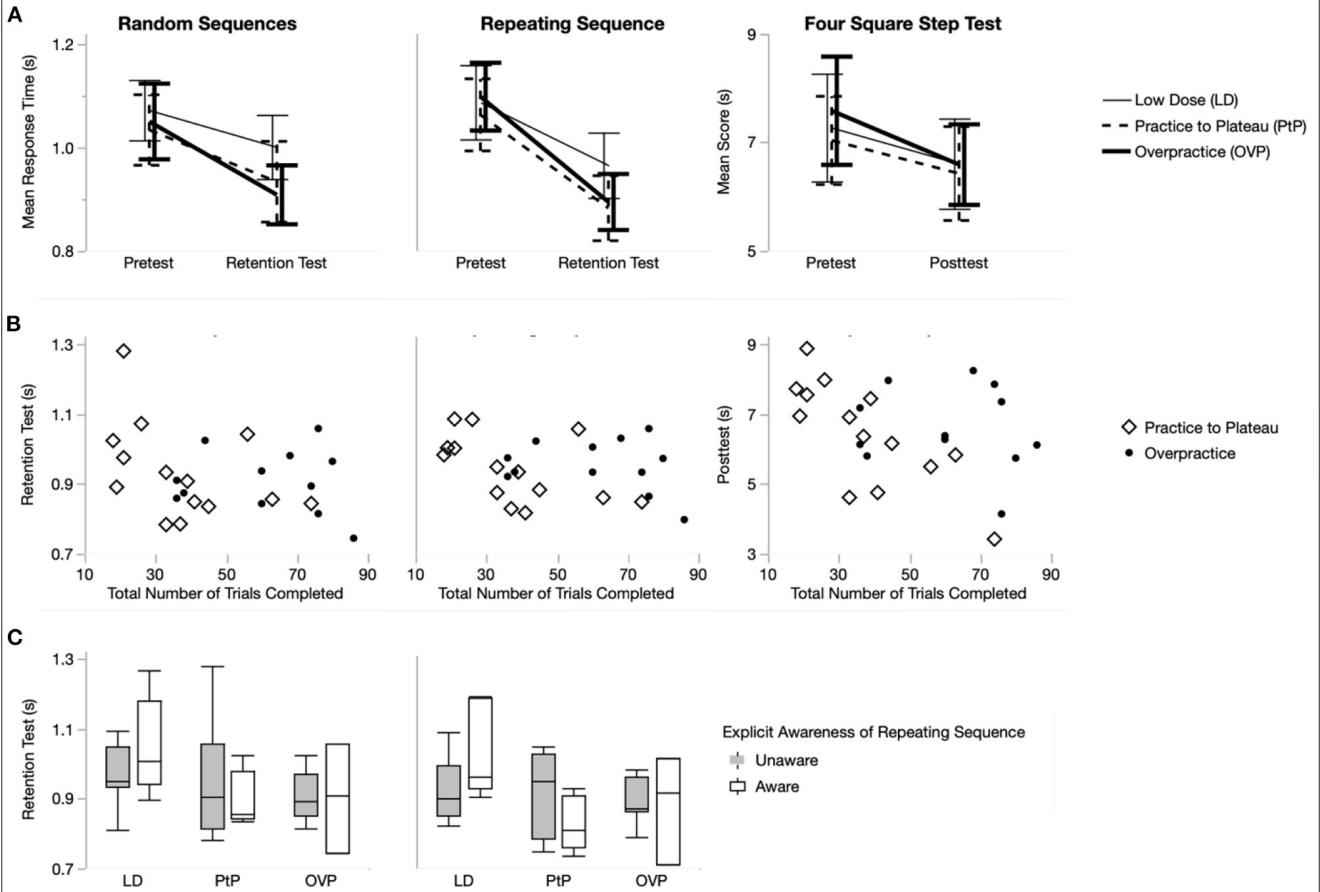
Each column is titled to indicate the dependent variable to which its figures correspond: Random Sequences (i.e., general learning), Repeating Sequence (i.e., repeated-sequence learning), and Four Square Step Test (i.e., transfer of learning). Row **(A)** shows the main effect of time for each of the dependent variables. Each plot shows mean response time (for the Serial Reaction Time Task) or mean time (for Four Square Step Test) at pretest and at retention test or posttest for each of the groups: LD (thin solid line), PtP (medium dashed line), and OVP (thick solid line). Error bars represent 95% confidence intervals. Row **(B)** shows separate scatterplots for each of the dependent variables. Each plot shows retention or posttest times as a function of the total number of trials of the practiced Serial Reaction Time Task for the two groups included in this analysis (note that the LD group is not included): PtP (open diamond) and OVP (solid circle). Row **(C)** shows box plots illustrating learning (at retention test) of the two serial reaction time task dependent variables: random sequences and repeating sequence. Each plot shows retention as a function of whether or not the participant became explicitly aware of the imbedded repeating sequence, and includes all three groups: LD, PtP, and OVP. LD, Low Dose group; PtP, Practice to Plateau group; OVP, Overpractice group.

Overall, participants demonstrated a strong general learning effect, as retention test response times on the random sequences were significantly faster than pretest times, *t*_(40)_ = −7.43, *p* < 0.001 (Figure 4A). The omnibus effect of group was not significant, although *post-hoc* testing showed significantly better general learning in the OVP compared to the LD group (*p* = 0.020; Table 2).

**Table 2:** Learning (i.e., retention) of the Serial Reaction Time Task (SRTT) and transfer effects on the Four Square Step Test (4SST).

**Descriptive statistics**				**Regression statistics** ^**†**^			
	**LD**	**PtP**	**OVP**	**Effect**	**Estimate**	**95% CI**	***p*-value**
**Random sequences**							
Pretest	1.07 (0.11)	1.03 (0.12)	1.05 (0.12)	Intercept	0.213	[-0.043, 0.469]	0.101
	[1.01, 1.13]	[0.96, 1.10]	[0.98, 1.12]	Pretest	0.735	[0.500, 0.970]	**<0.001**
Retention test	1.00 (0.11)	0.93 (0.14)	0.91 (0.09)	Group	–	–	0.063
	[0.94, 1.06]	[0.85, 1.01]	[0.85, 0.97]	(LD v. PtP)	−0.039	[−0.100, 0.022]	0.206
				(LD v. OVP)	−0.765	[−0.829, −0.701]	**0.020**
**Repeating sequence**							
Pretest	1.09 (0.13)	1.06 (0.12)	1.10 (0.10)	Intercept	0.212	[0.014, 0.410]	**0.036**
	[1.02, 1.16]	[0.99, 1.13]	[1.03, 1.17]	Pretest	0.693	[0.514, 0.872]	**<0.001**
Retention test	0.97 (0.11)	0.88 (0.11)	0.89 (0.09)	Group	–	–	**0.006**
	[0.91, 1.03]	[0.82, 0.94]	[0.84, 0.94]	(LD v. PtP)	−0.066	[−0.115, −0.017]	**0.010**
				(LD v. OVP)	−0.079	[−0.130, −0.028]	**0.004**
**Transfer task (4SST)**							
Pretest	7.28 (1.79)	7.04 (1.41)	7.58 (1.57)	Intercept	1.901	[0.784, 3.018]	0.065
	[6.29, 8.27]	[6.23, 7.85]	[6.58, 8.58]	Pretest	10.696	[10.551, 10.841]	**<0.001**
Posttest	6.61 (1.50)	6.43 (1.50)	6.59 (1.17)	Group	–	–	0.588
	[5.78, 7.44]	[5.56, 7.30]	[5.85, 7.33]	(LD v. PtP)	−0.005	[−0.539, 0.529]	0.996
				(LD v. OVP)	−0.927	[−1.484, −0.370]	0.360

Descriptive statistics of the time taken to complete each task are presented as mean (SD) [95% confidence interval].

† For the regression statistics, the Low Dose (LD) group served as the reference for the group effects.

PtP, Practice to Plateau group; OVP, Overpractice group; CI, Confidence Interval; 4SST, Four square step test.

**Bold** *p*-values indicate statistically significant effects, based on regression analyses and an alpha level of 0.05.

#### Repeated-sequence learning

Overall, participants demonstrated a strong repeated-sequence learning effect, as retention test response times on the repeating sequence were significantly faster than pretest times, *t*_(40)_ = −9.46, *p* < 0.001 (Figure 4A). The omnibus effect of group was significant (*p* = 0.006), and *post-hoc* testing showed that the PtP and OVP groups both demonstrated significantly better repeated-sequence learning compared to the LD group (*p* = 0.010 and *p* = 0.004, respectively; Table 2). However, there was no statistical difference in repeated-sequence learning between the PtP and OVP groups (*p* = 0.633).

#### Transfer of learning

Overall, participants improved on the transfer task despite having not explicitly practiced it, as times on the Four Square Step Test at posttest were significantly faster than those at pretest, *t*_(40)_ = −6.01, *p* < 0.001 (Figure 4A). The omnibus effect of group was not significant (*p* = 0.588; Table 2).

### Impact of the number of practice trials when controlling for group assignment

#### General learning

For general learning, participants who completed more practice trials were faster at retention, indicated by a negative relationship between the number of trials completed and random sequence retention test times, *t*_(23)_ = −2.17, *p* = 0.041, controlling for pretest (Figure 4B). However, after controlling for group assignment (PtP and OVP), this effect was attenuated and no longer statistically significant (*p* = 0.097) nor was there a statistically significant effect of group (*p* = 0.991; Table 3).

**Table 3:** Relationship of group assignment and number of trials completed to learning of the Serial Reaction Time Task (SRTT) and transfer effects on the Four Square Step Test (4SST).

**Dependent variable**	**Effect**	**Estimate**	**95% CI**	***p*-value**
Random sequences	Intercept	0.253	[−0.055, 0.561]	0.103
	Pretest	0.715	[0.433, 0.997]	**<0.001**
	Total trials	−0.002	[−0.003, 0.0003]	0.097
	Group	−0.0004	[−0.078, 0.077]	0.991
Repeating sequence	Intercept	0.329	[0.056, 0.602]	**0.020**
	Pretest	0.587	[0.343, 0.831]	**<0.001**
	Total trials	−0.002	[−0.003, −0.0003]	**0.020**
	Group	0.036	[−0.030, 0.101]	0.270
Transfer (4SST)	Intercept	1.947	[0.083, 3.810]	**0.041**
	Pretest	0.717	[0.508, 0.926]	**<0.001**
	Total trials	−0.015	[−0.033, 0.002]	0.089
	Group	0.127	[−0.597, 0.852]	0.719

Note that the Practice to Plateau (PtP) group served as the reference condition for the effect of Group, and the Low Dose (LD) group was not included in these analyses.

OVP, Overpractice group; CI, Confidence Interval; 4SST, Four square step test.

**Bold** *p*-values indicate statistically significant effects, based on regression analyses and an alpha level of 0.05.

#### Repeated-sequence learning

For repeated-sequence learning, participants who completed more practice trials were faster at retention, indicated by the negative relationship between the number of trials completed and repeating sequence retention test times, *t*_(23)_ = −2.26, *p* = 0.034, controlling for pretest (Figure 4B). After controlling for group assignment (PtP and OVP), this effect remained statistically significant (*p* = 0.020) but the effect of group was not (*p* = 0.270; Table 3).

#### Transfer of learning

On the Four Square Step Test, participants in this sample who completed more practice trials tended to be faster at posttest, however, this negative relationship between the number of trials completed and posttest times was not statistically significant, *t*_(23)_ = −2.04, *p* = 0.053, controlling for pretest (Figure 4B). When we controlled for group assignment (PtP and OVP), this effect was further attenuated (*p* = 0.089), and there was no statistically significant effect of group (*p* = 0.719; Table 3).

### Impact of explicit awareness

#### General learning

Explicit awareness did not influence general learning, evidenced by no significant main effect of explicit awareness (*p* = 0.305), nor a group-by-explicit awareness interaction (*p* = 0.462) (Figure 4C).

#### Repeated-sequence learning

For repeated-sequence learning, there was no significant main effect of explicit awareness (*p* = 0.071); however, there was a group-by-explicit awareness interaction (*p* = 0.022) (Figure 4C). *Post-hoc* testing revealed that, controlling for pretest, participants in the LD group who were explicitly aware had slower response times at retention test compared to those who were not aware (*p* = 0.033). In the PtP (*p* = 0.074) and OVP groups (*p* = 0.728), there were no statistically significant effects of explicit awareness.

## Discussion

Typically, investigations of practice dosages assign all participants in a group to the exact same number of practice repetitions as one another. However, given the inter-individual variability of motor skill acquisition and learning (13, 14), more personalized methods of dosing may be necessary. This study sought to investigate one such personalized method, in which practicing to or beyond one's individual practice plateau differentiates and standardizes the groups' practice dosages. First, our findings demonstrate that older adults can tolerate high and personalized dosages of postural training delivered through a standing stepping task, as none of our participants experienced any adverse events. Second, participants collectively demonstrated general learning, repeated-sequence learning, and transfer of learning. For both general and repeated-sequence learning, at least one of the personalized practice dosages resulted in more learning than the LD group, but the two personalized dosages did not differ from one another (although the study was not powered for this analysis). Third, for transfer of learning, the three groups did not perform differently from one another. In sum, these findings suggest that personalizing practice dosages based on a practice plateau may improve learning compared to practice dosages more similar to those provided in standard clinical care (5, 6). Our finding that total number of practice trials performed was more strongly related to repeated-sequence learning than was group assignment reinforces previous work showing that more practice is generally better (4).

A clear finding from this study was that LD practice dosages were inferior to higher practice dosages when it came to learning of the practiced task, which is also a common and important finding in neurorehabilitation studies (4, 47). Our LD group performed a very low dose of practice relative to the personalized practice groups, and their inferior retention could be attributed to under-dosing of practice. Ironically, to ensure the LD group was not under-dosed compared to actual standard-of-care in physical rehabilitation, we chose a practice dosage (144 total steps taken, with 72 serving as pretest and 72 serving as practice) well-beyond what is reported in studies describing current clinical practice which show that typical physical and occupational therapy sessions include very few repetitions of purposeful task practice addressing balance and postural control deficits (averaging 27 repetitions per session) (5, 6). The poor learning of our LD group, despite being dosed higher than what is seen in clinical physical rehabilitation settings, further implicates standard-of-care dosing and the need to raise this standard, particularly since the rates of learning tend to be even slower in aging and individuals with neurological conditions (48–50).

While this was a pilot study that was powered *a priori* to find an omnibus effect of group, it is worth noting that we did not find evidence that overpractice was better for learning than was stopping practice upon reaching plateau (i.e., no differences between PtP and OVP in primary analyses). This was especially true when accounting for the difference in total number of trials between groups (i.e., secondary analyses, which controlled for total number of trials performed). As such, the current data suggest that the absolute amount of practice was a stronger determinant of learning than whether that amount went beyond an individual's calculated plateau. Nevertheless, it is noteworthy that our two personalized groups achieved a high dose of practice because they were aiming to practice to and beyond their personal plateaus, respectively. If more practice does indeed optimize motor learning, the question remains “how much more?” While this study does not directly answer that question, its findings do suggest that seeking to reach or exceed the repetitions required to accomplish a practice plateau is one systematic method for achieving a high practice dosage, and may be more appropriate than arbitrarily choosing a number of repetitions that is simply assumed to be “high dose.”

To our knowledge, this is the first time this specific personalized practice dosage paradigm has been studied. Thus, this study is critical for first testing its efficacy and feasibility in individuals without frank neurologic deficits prior to exploring its utility in other populations, which is why we included community-dwelling older adults with no specific diagnosis and a range of balance impairment. Consistent with age-related postural control decline, our cohort demonstrated measurable postural control impairments, as evident by mini-Balance Evaluation Systems Test scores. This finding is expected, as balance impairment and falls are common, dangerous, and costly among older adults (51), including those living independently in the community, emphasizing the salience of studying methods to improve postural motor learning in the general older population.

### Limitations and future directions

The authors acknowledge the small sample size, although it was sufficient to find an omnibus effect of group for repeated-sequence learning. Although this study did not find differences between our two personalized practice dosages, it was not powered *a priori* to do so. Additionally, in this sample, on average the PtP group required more practice trials than the OVP group to reach a plateau, suggesting an unaccounted-for difference between the two groups that may have impacted learning. Future studies should be powered to test for a difference between these two personalized practice dosages.

It is worth noting that our assessors were not blinded to group assignment at retention and post-testing. To mitigate this limitation, assessors read a script verbatim throughout the assessment session, and were instructed not to deviate from nor add to it. Additionally, the primary outcomes data were collected by the instrumented step mat and computer, which were not able to be changed nor influenced by the human assessors.

While our calculation of the point of plateau appeared to successfully capture the time during practice when our participants reached their performance plateau, this was the first study to use this method. More well-established methods were first considered during initial pilot testing, such as three-parameter exponential decay functions that were applied to existing datasets collected using the same motor task (20, 33). However, multiple barriers were encountered that prevented this from being a viable approach. For example, a proportion of learners' data were poorly fit by the exponential decay function, with some models failing to converge. Additionally, when used iteratively and prospectively, the parameter estimates from the exponential decay function were extremely unstable, such that the predicted point of plateau after trial 20, for example, was dramatically different from the predicted value after trial 21. We observed that trial-by-trial, the parameter estimates would swing drastically, rendering them useless for prospective predictions, and resulting in our selection of an alternative novel method for defining plateau. Ultimately, the value of our primary findings hinges critically on our chosen method's ability to identify plateau. Thus, future work should formally assess the validity of this method in various populations and investigate other methods for prescribing personalized practice dosages to optimize motor learning.

## Data availability statement

The datasets presented in this article are not readily available because the raw data supporting the conclusions of this article will be made available by the authors upon request, as allowable by the University of Utah, including its Institutional Review Board. Requests to access the datasets should be directed to g.olivier@utah.edu.

## Ethics statement

The studies involving human participants were reviewed and approved by University of Utah Institutional Review Board. The patients/participants provided their written informed consent to participate in this study.

## Author contributions

GO, SS, LD, SP, KL, KF, KD, and HH: concept/idea/research design. GO, SS, and LD: drafting the manuscript and funding procurement. GO: data collection. KL, GO, and RM: data analysis. GO: participant recruitment and project management. LD: facilities/equipment and administrative support. All authors critically revising the manuscript for important intellectual content and final approval of the submitted manuscript.

## Funding

This work was partially supported by the Undergraduate Research Opportunities Program at the University of Utah in Salt Lake City, Utah, and the National Institutes of Health (K01 AG047926). The funding sources were not involved in any of the following: study design; collection, analysis, or interpretation of data, writing the report; nor submitting the article for publication.

## Conflict of interest

The authors declare that the research was conducted in the absence of any commercial or financial relationships that could be construed as a potential conflict of interest.

## Publisher's note

All claims expressed in this article are solely those of the authors and do not necessarily represent those of their affiliated organizations, or those of the publisher, the editors and the reviewers. Any product that may be evaluated in this article, or claim that may be made by its manufacturer, is not guaranteed or endorsed by the publisher.

## Footnotes

^1^Note that Median Response Time was used for providing feedback to participants. Feedback was provided in real-time, based on the raw data, prior to “cleaning” the data of outliers related to technical difficulties. The authors preferred this measure of central tendency for providing feedback, because the Median is more resistant to outliers. In contrast, Mean Response Time was used as the measure of central tendency for data analysis, because at that point the outliers had already been removed (see Section Data Reduction and Statistical Analyses for more details).

^2^Note that the section entitled *Defining Practice Plateau* (which describes the real-time calculation of practice plateau) reports a more conservative threshold for outliers (i.e., >5 s). The 5-s value was chosen for this purpose because the algorithm was designed to calculate practice plateau in real-time and was developed *a priori*, without the benefit of knowing how this specific cohort would perform on the task. Instead, selection of the 5-s outlier value was informed by previous cohorts who have performed this task, which included patients with Parkinson disease, who move more slowly than a “general” older adult population. We were committed to selecting a conservative value, which would avoid inadvertently excluding response times that reflected true participant performance and errors. In contrast, *post-hoc* data reduction was informed by the complete dataset as well as staff notes taken during participant sessions, which itemized technical difficulties that arose. during testing and training (e.g., step mat time-outs, computer crashing). Cross-referencing data with staff notes made it clear that, in the absence of technical errors, response times were consistently <2.5 s in this cohort. Thus, with the benefit of more information, we chose a more appropriate outlier threshold (i.e., 2.5 s), in order to exclude true outliers due to technical difficulties.

^3^*Transfer of learning* was defined as posttest performance on the Four Square Step Test. Planned analyses, published during trial registration, included change scores for each of these three outcomes measures; however, prior to analysis, this plan was modified in favor of the more statistically-sound approach of using retention (or posttest) performance as the dependent variable while controlling for pretest performance (43).
